# A large tyrannosaurid from the Late Cretaceous (Campanian) of North America

**DOI:** 10.1038/s41598-026-38600-w

**Published:** 2026-03-12

**Authors:** Nicholas R. Longrich, Sebastian Dalman, Spencer G. Lucas, Anthony R. Fiorillo

**Affiliations:** 1 Department of Life Sciences, University of Bath, Bath, BA2 7AY UK; 2 Montana State University, Missoula, MT USA; 3 New Mexico Museum of Natural History and Science, 1801 Mountain Road N.W, Albuquerque, NM 87104 USA

**Keywords:** Ecology, Ecology, Evolution, Zoology

## Abstract

The Tyrannosauridae emerged as the dominant large predators in Laurasia during the Late Cretaceous. Their evolution in North America culminated with the replacement of Albertosaurinae, Daspletosaurinae, and Teratophonei, with masses of 2-3 tonnes, by the giant *Tyrannosaurus* in the late Maastrichtian, with a mass approaching or exceeding 10 tonnes. The origin of *Tyrannosaurus* remains enigmatic, but fossils suggest an origin in the Campanian—Maastrichtian of southern Laramidia. Here we describe an unusually large and robust tyrannosaurid tibia from the late Campanian aged Hunter Wash Member of the Kirtland Formation, New Mexico, ~ 74 Ma. The tibia measures 960 mm in length and 128 mm in diameter, ~84% and 78% the dimensions of the largest known *Tyrannosaurus*, suggesting a mass approaching 5 tonnes, larger than any contemporary tyrannosaur. The Hunter Wash tyrannosaur could potentially represent (i) an unusually large and robust *Bistahieversor*, (ii) a previously unknown lineage of giant tyrannosaur, or (iii) an early representative of Tyrannosaurini. The tibia’s large size, robust proportions, and shape of the distal shaft are most consistent with referral to Tyrannosaurini. The Hunter Wash tyrannosaur emphasizes the marked endemicity of Laramidian dinosaurs; while smaller Albertosaurinae and Daspletosaurini inhabited the north, giant tyrannosaurins occurred in the south.

**Supplementary Information:**

The online version contains supplementary material available at 10.1038/s41598-026-38600-w.

The Tyrannosauridae were among the last and the largest of the predatory dinosaurs^1–3^. Following the extinction of the carcharodontosaurs in the mid-Cretaceous, tyrannosaurs diversified and evolved large size, becoming the dominant predators of the latest Cretaceous in both North America and Asia^1–5^. By the late Campanian, multiple groups of tyrannosaurids, including Albertosaurinae, Daspletosaurini, and Teratophonei, had achieved large sizes of 2–3 tonnes^6,7^. Their evolution culminated in the appearance of the giant *Tyrannosaurus*^4,8,9^, both one of the last tyrannosaurids and the largest tyrannosaurid^10^, and perhaps the largest known predatory dinosaur ever to evolve^6,7^.

The origin of *Tyrannosaurus* remains unclear. Some evolutionary scenarios suggest a possible immigration from Asia^2,5,11,12^, whereas other scenarios suggest an origin in North America^13–15^. The identification of the giant *Tyrannosaurus mcraeensis*^13^ from the upper Campanian or lower Maastrichtian of New Mexico as the sister taxon of *T*. *rex*^5,13^, together with mid-Maastrichtian *Tyrannosaurus* sp. in Texas^16,17^, favor a southern Laramidian origin of *Tyrannosaurus*, followed by dispersal of the genus northward in the late Maastrichtian^13^. However, the origin of *Tyrannosaurus* remains debated^11^.

The southern origins hypothesis makes a testable prediction about the fossil record: if *Tyrannosaurus* evolved in southern North America, further study of the fossil record should reveal other evidence of early Tyrannosaurini in southern Laramidia. Here, we report a giant tyrannosaur dating to approximately 74 Ma, from the late Campanian of New Mexico. This represents the oldest known giant tyrannosaur from North America and may represent the oldest known member of the Tyrannosaurini.

Systematic Paleontology.

Dinosauria— Owen, 1842.

Theropoda— Marsh, 1881.

Coelurosauria— von Huene, 1914.

Tyrannosauridae— Osborn, 1905.

Tyrannosaurinae— Currie, 2003.

cf. Tyrannosaurini— Olshevsky, 1995.

Tyrannosaurini indet.

## Horizon and locality

**Fig. 1:**
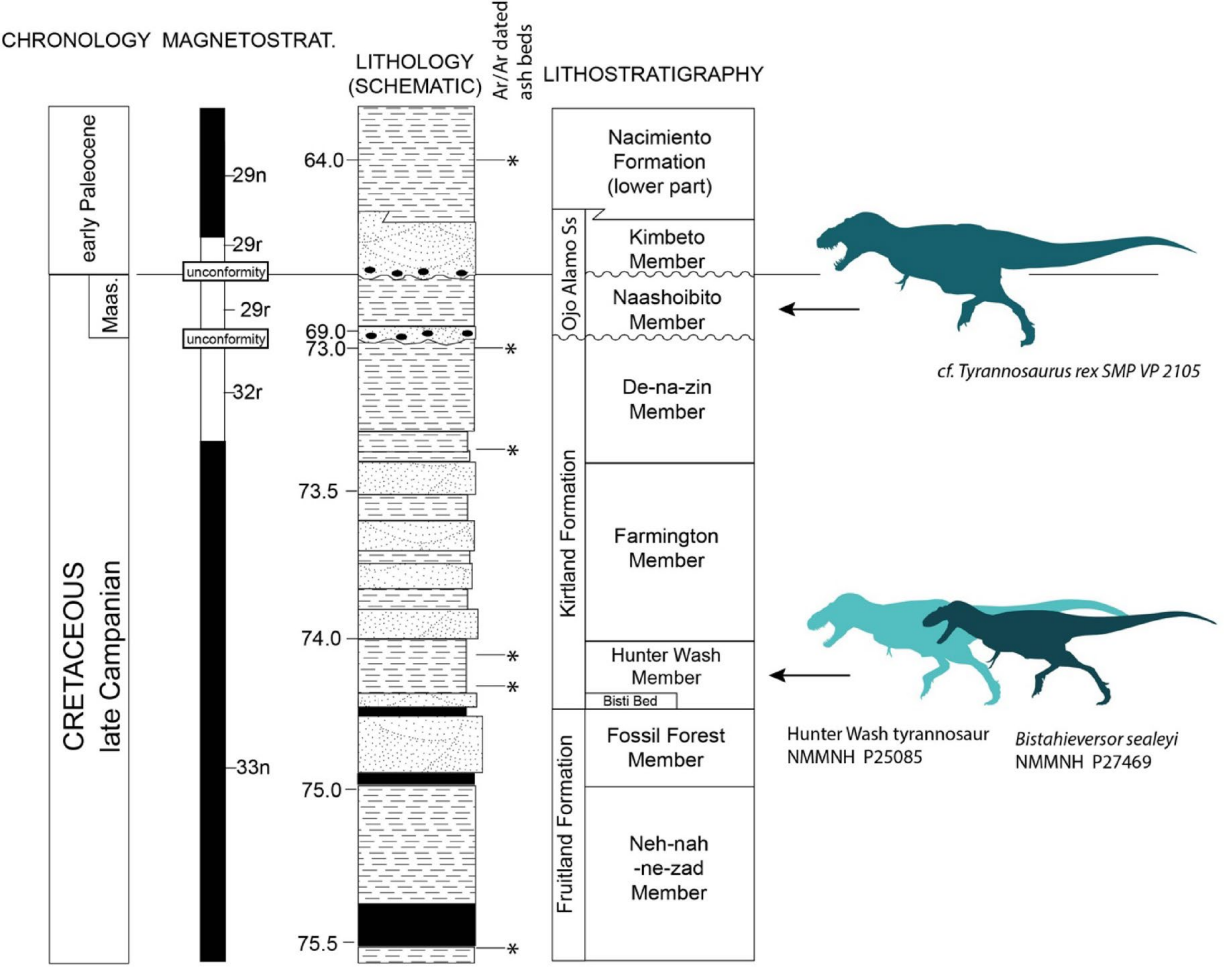
Stratigraphy of the San Juan Basin showing stratigraphic position of the Kirtland Formation giant tyrannosaur, NMMNH P-25085. The specimen comes from the Hunter Wash Member of the Kirtland Formation. Ar/Ar radiometric dating of ash beds suggest an age of ~ 74 Ma for the Hunter Wash Member.

NMMNH locality 1633, Hunter Wash Member, Kirtland Formation, San Juan Basin, New Mexico^18^. NMMNH P-25085 was originally stated to have come from the De-Na-Zin Member of the Kirtland Formation^18^, but the original field and database records at NMMNH indicate it was collected just southeast of Alamo Mesa (and northwest of Hunter Wash), in strata of the lower member of the Kirtland Formation, the Hunter Wash Member^19^. The Hunter Wash Member has yielded a vertebrate fauna of the Kirtlandian land-vertebrate age, which is Campanian. Magnetostratigraphically its strata are of normal polarity and assigned to chron 33n, which is Campanian^20^. Furthermore, Ar/Ar dating of ash beds in the Hunter Wash Member indicate ages of ~ 74 Ma ^20,21^ to ~ 75 Ma^22^. An ash bed in the underlying Fruitland Formation has an Ar/Ar age of about 76 Ma 22 to 75.166^22^. The stratigraphically higher De-Na-Zin Member of the Kirtland Formation has an ash bed Ar/Ar dated to a little older than 73 Ma to 73.496 Ma^22^. We can thus say that biostratigraphic, magnetostratigraphic and radiometric data and analyses agree that the Hunter Wash Member is late Campanian, about 74–75 Ma, and that is the age of the tyrannosaurid tibia, NMMNH P-25085. It is therefore slightly younger than the faunas known from uppermost Dinosaur Park Formation of Alberta, the Judith River Formation of Montana, or the fossiliferous part of the Kaiparowits Formation, and penecontemporaneous with the uppermost Two Medicine Formation^22^. It is slightly older than the dinosaurs known from either the Hall Lake Formation of New Mexico^13^, the Cerro Del Pueblo Formation of Mexico^15^, or the Horseshoe Canyon Formation of Alberta^23^ (Fig. 1).

## Material

NMMNH P-25085, left tibia.

### Description and comparisons

**Fig. 2:**
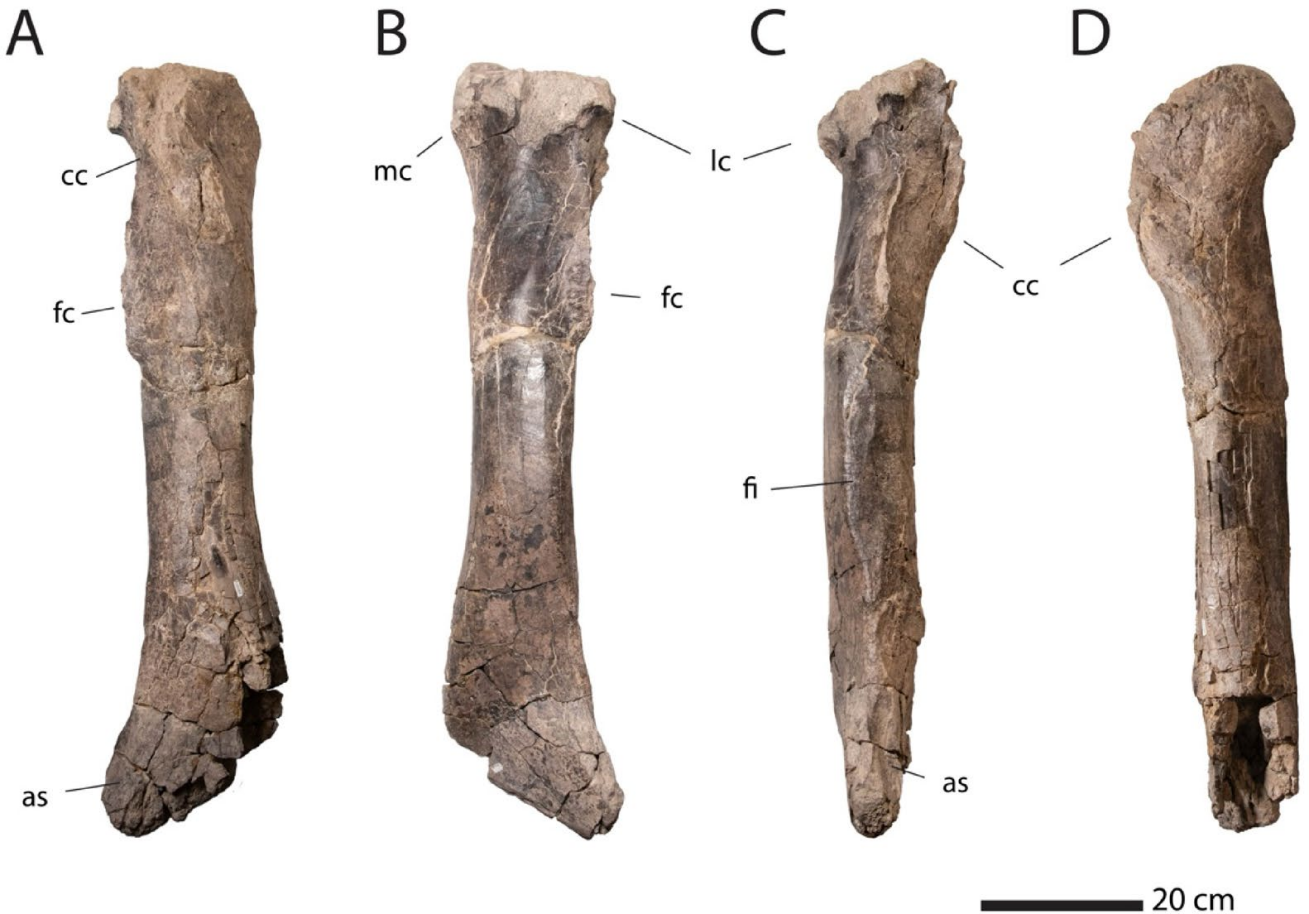
Hunter Wash tyrannosaurid NMMNH P-25085, left tibia in **A**, anterior, **B**, posterior, **C**, lateral, and **D**, medial. Abbreviations: as, astragalus; cc, cnemial crest; fc, fibular crest; fi, fibular facet; lc, lateral condyle; mc, medial condyle.

**Fig. 3:**
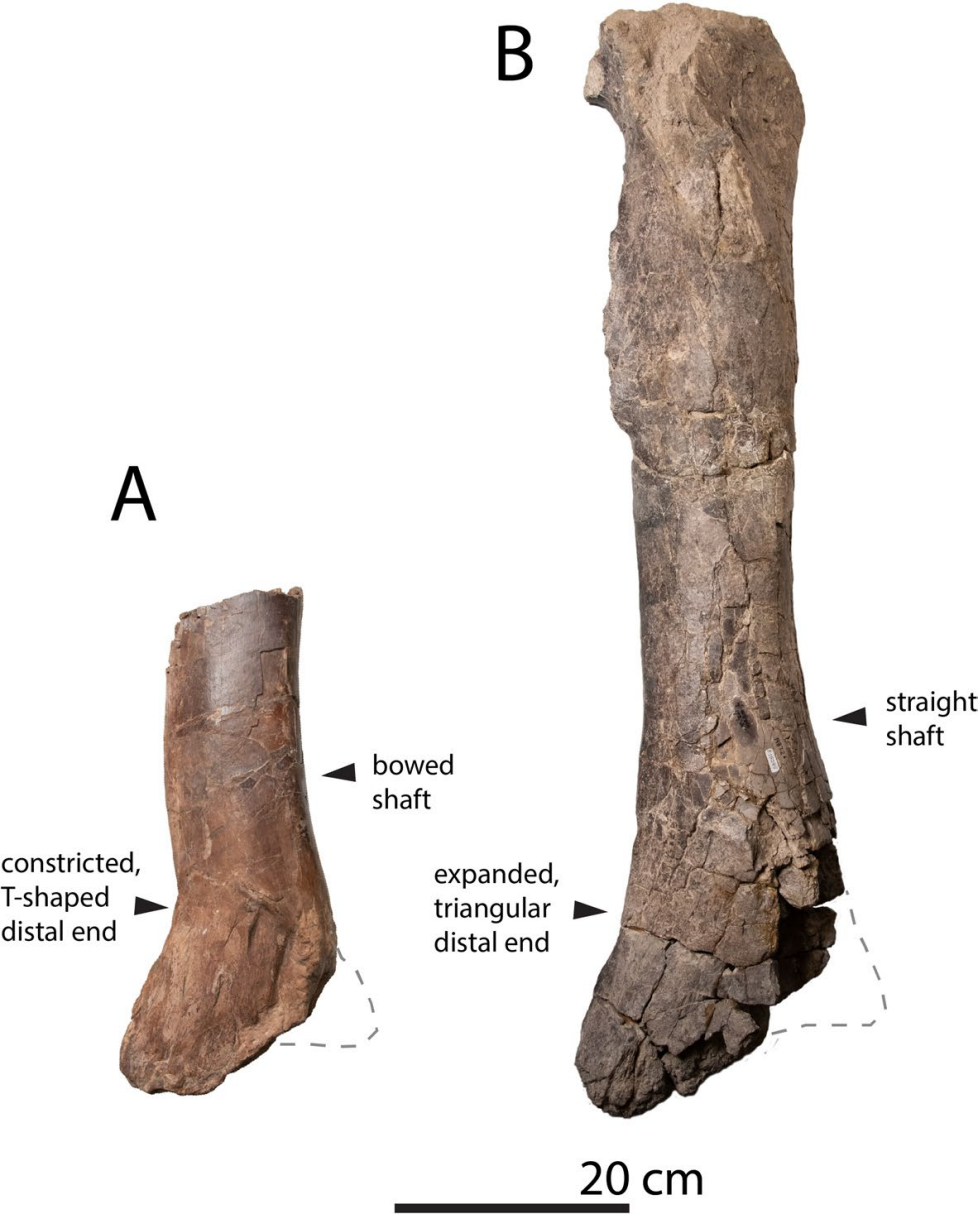
(**A**), cf. *Bistahieversor sealeyi* OMNH 10131, (**B**) NMMNH P-25085, Hunter Wash of the Kirtland Formation. Note the straighter shaft and broadly expanded, triangular distal tibia in the Hunter Wash tyrannosaurid.

Description. NMMNH P-25085 (Fig. 2) is a large and robust tyrannosaur tibia^18^. NMMNH P-25085 is complete except for damage to the proximal condyles and to the medial malleolus of the distal end. It measures 960 mm in length. Its mediolateral diameter is 128 mm, 13.3% of tibia diameter. By comparison, the tibia of “Sue” FMNH PR2081, measures 1143 mm long and has a midshaft width of 165 mm^24^, 14.4% tibia diameter. The anteroposterior diameter of NMMNH P-25085 is 110 mm, and the distal width is 200 mm (Fig. 3).

**Fig. 4:**
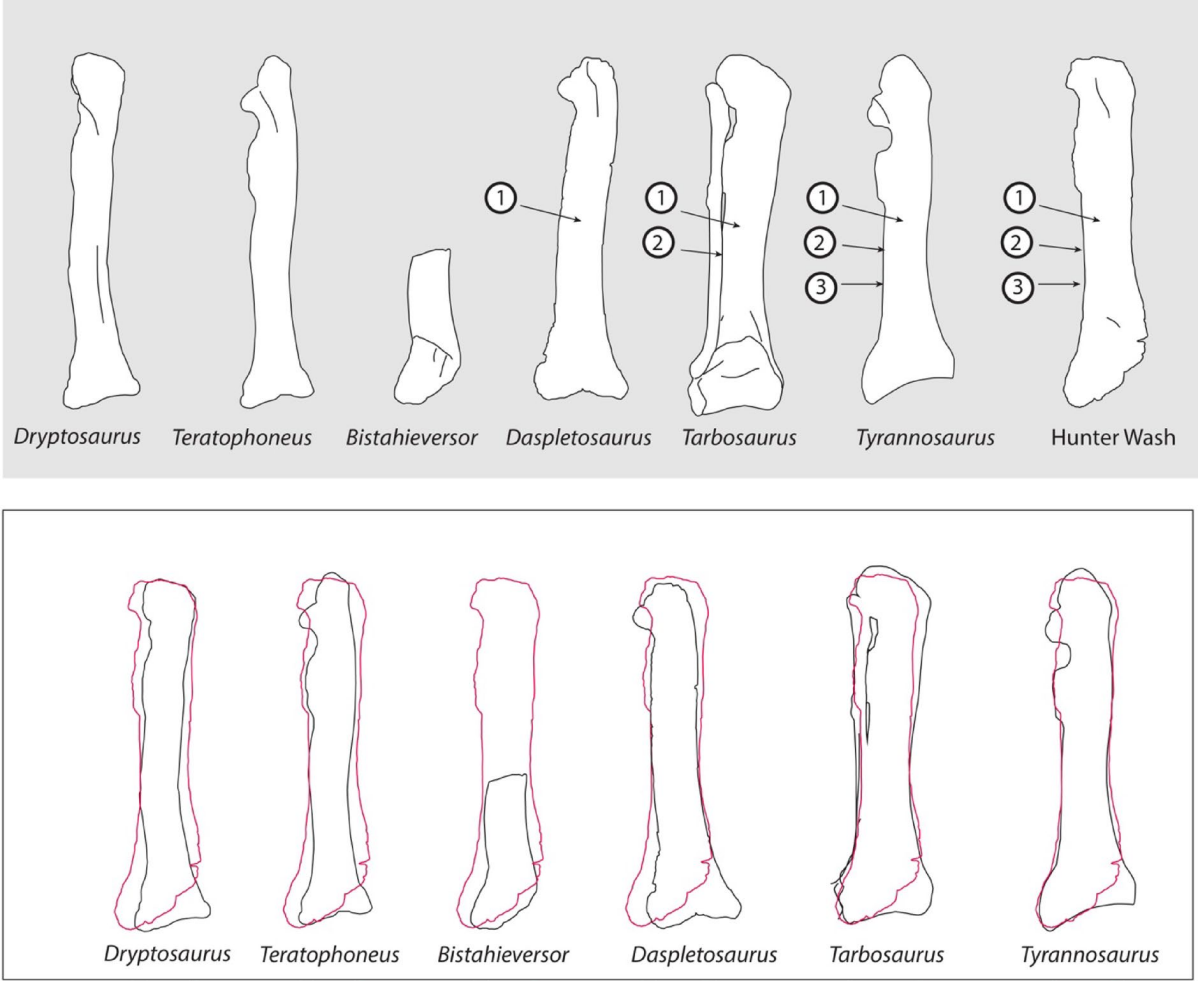
NMMNH P-25085, Hunter Wash of the Kirtland Formation, compared to *T. rex* and other Tyrannosauridae. The robust construction, straight shaft, and strongly triangular distal expansion of the tibia NMMNH P-25085 are all shared with *Tyrannosaurus* to the exclusion of other North American tyrannosaurs, including cf. *Bistahieversor* OMNH 10131. Top, derived features of NMMNH P-25085 and their distribution in Tyrannosauridae: (1) robust tibial shaft, (2) straight tibia shaft, (3) distal end of tibia with a long, triangular expansion. From left to right: *Dryptosaurus aquilunguis*^28^, *Teratophoneus curriei*^14^, cf. *Bistahieversor sealeyi* (OMNH 10131), *Daspletosaurus horneri*, *Tarbosaurus bataar*^26^, *Tyrannosaurus rex*^24^, Hunter Wash tyrannosaurid NMMNH P-25085. Below: outline of NMMNH P-25085 (red) superimposed on other tyrannosaurs.

**Fig. 5:**
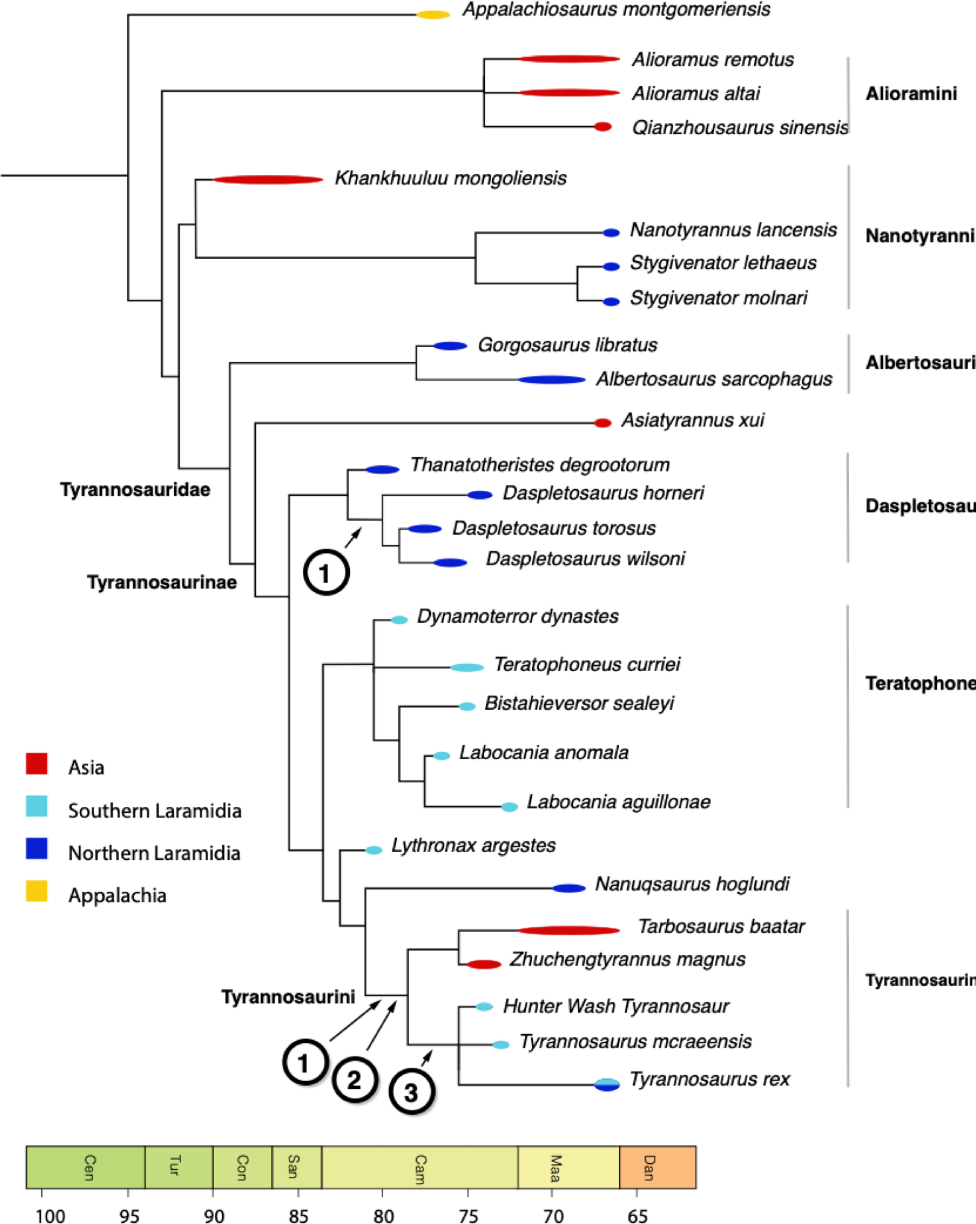
Affinities of the Hunter Wash tyrannosaurid. Strict consensus of 1404 most parsimonious trees, equal-weights parsimony. NMMNH P 25085 is recovered as part of the Tyrannosaurini, in an unresolved polytomy with *Tyrannosaurus mcraeensis* and *Tyrannosaurus rex*. Characters supporting placement of NMMNH P 25085: (1), robust hindlimb bones (tibia diameter ≥ 12% tibia length), (2) straight tibia, (3) distal end of tibia shaft expanded and triangular.

The tibia differs markedly in shape from that of *Bistahieversor* OMNH 10131 (Fig. 4) and closely resembles that of *Tyrannosaurus rex* (Fig. 5).

The proximal condyles are massive and separated posteriorly by a narrow groove. There is a robust cnemial crest that projects anterolaterally; its end is damaged. The fibular crest is subrectangular, similar to the condition in *T*. *rex*^24^, and unlike the more rounded fibular crest seen in basal tyrannosaurids such *Teratophoneus*^14^ and *Albertosaurus*^25^. It is not as laterally extended as in *T*. *rex*^24^, and it also extends more proximally. There is a broad, flat facet on the tibia’s lateral surface where the fibula made a ligamentous contact.

The lateral margin of the tibia shaft is almost straight, as in *Tyrannosaurus*^24^ and *Tarbosaurus*^26^ (Fig. 4). This contrasts with the bowed condition in more primitive tyrannosaurids, including *Daspletosaurus*, *Lythronax*^14^, *Teratophoneus*^14^, *Bistahieversor*^27^, and *Albertosaurus*^25^. The tibia is also strongly bowed in the basal tyrannosauroid *Dryptosaurus*^28^.

The Hunter Wash tibia is also remarkably robust in either anterior or lateral view. This is a derived feature shared with *Tyrannosaurus*^24^ and *Tarbosaurus*^26^, as well as *Daspletosaurus*. The tibiae of more primitive tyrannosaurids including *Lythronax*^14^, *Teratophoneus*^14^ and *Albertosaurus*^25^ are relatively slender, as are the tibiae of basal tyrannosauroids such as *Dryptosaurus*^28^ and Alioramini^29^.

Distally, the tibia gradually flares out, giving the distal end of the bone a long, broadly triangular shape. This triangular expansion extends about 25% the length of the entire tibia^24^. Although *Daspletosaurus* has a triangular expansion, it is relatively shorter, only about 20% the length of the tibia shaft, and so more primitive than the condition in *Tyrannosaurus*^24^ and NMMNH P-25085.

The only other tyrannosaur known to show a long, triangular expansion of the tibial shaft is *Tyrannosaurus*^24^. In all other known Tyrannosauridae, the shaft of the tibia is narrow distally just above the astragalar condyles, then the medial and lateral malleoli flare out, giving the bone a sort of T-shape in cross section (Fig. 4).

### Comparisons

NMMNH P-25,085 can be referred to Tyrannosauridae based on its large size, the strongly triangular medial malleolus and the strongly angled contact of the distal end of the tibia with the calcaneum and astragalus.

The robustness of the tibia is approached by *Daspletosaurus*, *Tarbosaurus* and *T. rex* (Fig. 4). The straight lateral margin of the tibia is a derived feature shared only by *Tyrannosaurus* and *Tarbosaurus* among known tyrannosaurids (Fig. 4). The very long, broad triangular distal expansion of the shaft is a derived feature uniquely shared with *Tyrannosaurus*. None of these features are seen in *Bistahieversor* OMNH 10131^27^ (Fig. 3) arguing against referral to *Bistahieversor*. Although NMMNH P-25085 is larger than OMNH 10131, the size difference does not seem sufficient for ontogeny to explain these differences. Furthermore, the robust postcranial skeleton seen in *Tyrannosaurus* is not simply a function of the large sizes achieved by Tyrannosaurini, because even the smallest known *Tyrannosaurus* specimens have robust hindlimb elements^30^ compared to similar-sized albertosaurines^25^ and nanotyrannosaurs^5^.

Therefore, at least four derived features— the robust tibia, the straight shaft, the triangular distal shaft, and the overall large size of the animal— are shared with *Tyrannosaurus* among North American tyrannosaurids. The available evidence suggests affinities with Tyrannosaurini or even that the tibia belongs to an early species of *Tyrannosaurus* itself.

### Mass estimation

Because limb bones are weight-bearing, their dimensions are tightly correlated with body mass and thus can be used to estimate body mass^6,31,32^. Regression equations have been used to estimate mass from limb dimensions in dinosaurs. Although published equations for bipeds focused on mass estimates using femur dimensions rather than the tibia^6,31,32^, there are several approaches we can use to estimate mass for NMMNH P-25085.

The simplest approach is to use isometric scaling. Linear measurements for a tyrannosaur specimen of unknown mass can be compared to the same measurement for specimens for which mass estimates are available (either from regressions of limb bone dimensions, or volumetric approaches). The Hunter Wash tyrannosaur has a tibia length of 960 mm, and the tibia shaft has a mediolateral diameter of 128 mm. By comparison, FMNH PR 2081 (“Sue”) has a tibia length of 1143 mm and a mediolateral diameter of 165 mm^24^. The tibia of NMMNH P-25085 is therefore 0.84 the length and 0.775 the diameter of FMNH PR 2081. Since volume and mass scale as the cube of linear dimensions, we arrive at estimates of 0.592 and 0.467 for the mass of NMMNH P-25085 relative to “Sue” FMNH PR 2081 by comparing length and diameter, respectively. The mass of FMNH PR 2081 itself has been the focus of many studies and is estimated at around 8,000–10,000 kg^10,33^ depending on the approach used. If we assume a mass of 9,500 kg^10^, this implies a mass for NMMNH P-25085 of 5629 kg based on tibial length, or 4435 kg based on tibial diameter. Isometric scaling ignores allometric effects, but allometric effects are largest when comparing animals of very different sizes. Errors are minimized by comparing animals of similar size (as done here).

Although no allometric calculations have been published for mass estimation using tibia diameter it still may be possible to attempt an allometric approach. Mass can be estimated from femur circumference using the equation log_10_M = 2.754 * log_10_C-0.683^32^. Since tibia diameter is proportional to circumference, mass can be approximated as proportional to the 2.754 power of diameter (assuming tibia diameter scales similarly to femoral diameter) rather than the third power, or 9500 kg *(0.84)^2.754^ using the 9.5 tonne estimate for FMNH PR 2081. This yields a mass of 4720 kg for NMMNH P-25085.

These estimates are of course based on masses for Sue, which are, themselves, estimates with their own potential errors. Larger or smaller estimates for Sue obviously produce larger or smaller estimates for NMMNH P-25085. Thus, assuming a 7.7 tonne mass for Sue^7^, then using isometric scaling, the Hunter Wash tyrannosaur has a mass of 3595 kg based on tibia diameter and 4562 kg based on tibia length; using allometric scaling with an exponent of 2.754 it has a mass of 3826 kg. Assuming a mass of 10 tonnes^33^ the Hunter Wash tyrannosaur is estimated to weigh 4668 kg (isometric tibia diameter), 5924 kg (isometric tibia length), and 4969 kg (allometric tibia diameter).

Again, our purpose is not to make a *precise* weight estimate for NMMNH P-25,085 but to show that using a variety of assumptions, the animal is reconstructed as unusually large relative to contemporary tyrannosaurs (Fig. 5).

Although the range of estimates show the uncertainty involved in mass estimation for NMMNH P-25085, this is true of all dinosaurs^10^. More importantly the estimates are consistently large— whether we estimate mass based on tibia length or tibia diameter, and using a variety of mass estimates for Sue, the Hunter Wash tyrannosaur is estimated as weighing at least 4 tonnes and perhaps as much as 5.9 tonnes; the most reliable estimate is perhaps the 4.7 tonne estimate using allometric scaling. This makes NMMNH P-25085 the largest tyrannosaur reported from the late Campanian thus far (Fig. 5).

In addition, animals naturally show a range of adult body masses due to environmental factors, genetics, and sexual dimorphism. Like living animals, individual dinosaurs in a population would have achieved different adult body masses, and it would be expected that a few grew to significantly larger size than the population average^34,35^. Among modern animals, large animals like lions, elephants and whales have a few world-record sized individuals^35^. It is possible— but improbable— that NMMNH P-25085 was one of the rare individuals from the upper end of its species body mass distribution. If it was an average member of its species, then larger individuals presumably existed. Further sampling of the Kirtland Formation could therefore reveal even larger tyrannosaurs in the Campanian.

### Phylogenetic analysis

NMMNH P-25085 was included in a previously published character-taxon matrix^15^ together with new characters designed to improve resolution of tyrannosaurid phylogeny, and specifically derived characters of the tibia to help constrain NMMNH P-25085. The resulting analysis includes 537 characters. *Khankhuuluu* *mongoliensis *was added^11^ along with four nanotyrannosaurs– *Nanotyrannus lancensis*, BMRP 2002.4.1 (holotype of “*Nanotyrannus*” *lethaeus*), NCSM 40,000, and LACM 28471(holotype of *Stygivenator molnari*). *Juravenator starkii* and *Scipionyx samniticus* appear to represent juveniles and so may not exhibit the derived features characterizing the adults of these species; both were excluded.

Phylogenetic analysis was conducted using PAUP* 4.0 b10 using equal weights parsimony. The analysis produces 1404 most-parsimonious trees of length 1925 (CI = 0.3673, RI = 0.7608) and an almost fully resolved strict consensus for Tyrannosauridae, with NMMNH P-25085 part of a clade containing *Tyrannosaurus rex* and *T*. *mcraeensis*. (Fig. 5). Use of implied weighting using a variety of concavity constants (K = 2, 6, 12) produced an identical placement for NMMNH P-25085. Forcing the Hunter Wash tyrannosaur outside *Tyrannosaurus* + *Tarbosaurus* requires two additional steps; forcing it below *Lythronax* requires an additional three steps.

## Discussion

The fragmentary nature of NMMNH P-25085 and most other tyrannosaurs known from the Fruitland and Kirtland Formations of New Mexico^18,27,36^ complicate attempts to infer relationships. However, isolated bones of dinosaurs and other vertebrates can often be diagnosed to a low taxonomic level, sometimes to genus or even species. Postcrania are rarely diagnostic to species level, but, even so, postcrania often have diagnostic characters^15,37^ allowing referral to a higher clade, e.g. subfamily or genus. The Hunter Wash tyrannosaur provides enough characters to attempt at least a preliminary identification.

There are three possible identifications of the Hunter Wash tyrannosaur:

(i) the Hunter Wash tyrannosaur represents an unusually large and robust individual of the tyrannosaurid *Bistahieversor sealeyi*^36^,
(ii) the Hunter Wash tyrannosaur represents a distinct and previously unrecognized lineage of tyrannosaurid unrelated to *Tyrannosaurus*, which independently achieved large size, or,
(iii) the Hunter Wash tyrannosaur represents an early member of Tyrannosaurini, the clade of large tyrannosaurids containing *Tyrannosaurus* and its Asian relatives *Tarbosaurus* and *Zhuchengtyrannus*.

*Bistahieversor sealeyi* is the only tyrannosaurid currently identified from the Hunter Wash Member^36^. Although the holotype does not preserve the limbs, OMNH 10131^27^, a partial tyrannosaur skeleton of cf. *Bistahieversor* from the Fruitland or lower Kirtland Formation, preserves the distal tibia. The tibia is smaller than that of the Hunter Wash tyrannosaur (Fig. 3) and differs in having a distinctly curved shaft, as well as a constriction of the shaft proximal to the distal end. The tibia’s mediolateral diameter is 113.5 mm and its circumference is 320 mm; using the same approach as outlined above, this corresponds to a mass of 3 tonnes. The femur of the animal measures 368 mm in circumference, which produces a somewhat lower mass estimate of 2.3 tonnes (although for our purposes the estimate from tibia diameter is more useful as it can be directly compared to the estimate for the Hunter Wash tibia).

Several lines of evidence therefore argue that the Hunter Wash tyrannosaur is distinct from *Bistahieversor*. First, *Bistahieversor* is a significantly smaller animal^36^; its mass has been estimated at approximately 2.5 tonnes^7^, about half the mass of the Hunter Wash tyrannosaur, and OMNH 10131 is here estimated at 2.3-3 tonnes. It is not impossible that *Bistahieversor* grew to significantly larger size, but the rugose skulls of the type and referred individual suggest they were near full size, and related taxa such as *Teratophoneus*^14,38^ and *Labocania*^15^ are not known to have grown larger.

Furthermore, as noted above, the *Bistahieversor* tibia (Fig. 3) differs in shape from the NMMNH P-25085 tibia in having a strongly curved tibial shaft and lacking a triangular distal expansion of the tibia. This argues for the existence of two distinct tyrannosaurs in the Fruitland-Kirtland succession— *Bistahieversor* and a second, larger, more robust and more derived form. It does not seem likely that these differences could reflect intraspecific and/or ontogenetic variation. Although it was previously believed that tyrannosaurids underwent radical morphological changes with age^39^, this is now known to reflect the lumping of distinct taxa^5,37^ which created the illusion that tyrannosaurid taxa are far more variable than they are, and postcrania have been shown to exhibit diagnostic features^5,37^. Assuming OMNH 10131 does belong to *Bistahieversor* this therefore precludes referral of NMMNH P-25085 to *Bistahieversor*.

Another possibility is that NMMNH P-25085 represents a previously unrecognized lineage of tyrannosaur, one that evolved large size independently of *Tyrannosaurus*. This hypothesis is difficult to reject definitely, but there is not much support for it. Support for this hypothesis would require showing that the anatomy of NMMNH P-25085 is inconsistent with any known tyrannosaur lineages including Tyrannosaurini, or else that its anatomy is more consistent with a known clade such as Albertosaurinae or Daspletosaurini. To our knowledge no features of NMMNH P-25085 either preclude referral to Tyrannosaurini, or support referral to any other tyrannosaur lineage.

The third and (in our assessment) best-supported hypothesis is that the Hunter Wash tyrannosaur is an early member of Tyrannosaurini, the clade including *Tyrannosaurus* and *Tarbosaurus*. Features supporting this assignment include (i) the animal’s large size, so far only seen in Tyrannosaurini among known tyrannosaurids, (ii) the extremely robust limb bones, again characteristic of Tyrannosaurini, although also found in *Daspletosaurus* and (iii) the relatively straight tibia, again characteristic of Tyrannosaurini. A fourth character, the expanded and triangular distal tibia shaft (iv), is uniquely shared with *Tyrannosaurus*. While hindlimb bones are typically less diagnostic at the species level than cranial elements, they are highly constrained by locomotion and likely to carry high phylogenetic signal.

Phylogenetic analysis recovers the Hunter Wash tyrannosaur in an unresolved polytomy with *Tyrannosaurus mcraeensis* and *Tyrannosaurus rex* (Fig. 5), supporting referral of NMMNH P-25085 to Tyrannosaurini or potentially even *Tyrannosaurus*. This topology is recovered in equal-weights parsimony and in implied weighting with different weighting schemes showing that the placement in Tyrannosaurini is robust against different character weightings. The NMMNH P-25085— *T*. *mcraeensis* — *Tyrannosaurus rex* clade has a 48% bootstrap, which is above the average 40% bootstrap support across all nodes in Tyrannosauridae.

We argue that referral to Tyrannosaurini is the most likely hypothesis because it is consistent with multiple lines of evidence: (i) anatomical evidence, including a morphological phylogenetic analysis, (ii) the unusual size of the animal, (iii) geography (early Tyrannosaurini, i.e. *Tyrannosaurus mcraeensis*, having previously been reported from the Southwest) and (iv) stratigraphy (existence of *Tyrannosaurus mcraeensis* in the latest Campanian or earliest Maastrichtian implies the existence of a ghost lineage extending into the late Campanian). While more complete remains may be needed to make a definitive identification of Tyrannosaurini in the Kirtland Formation, these different lines of evidence are all consistent with this hypothesis.

Regardless of which hypothesis is adopted, the unusual size of the Hunter Wash tyrannosaur is significant, as it represents a previously unrecognized appearance of large tyrannosaurids in the late Campanian, and shows that they evolved earlier than previously believed. The precise age of *Tyrannosaurus mcraeensis* remains unknown because radiometric dates constrain the age of underlying beds but not overlying strata^13^, it could therefore be latest Campanian or possibly early Maastrichtian in age^13^; the age of NMMNH P-25085 is well-constrained by radiometric dates above and below the Hunter Wash Member (Fig. 1) and therefore provides an important datapoint for the appearance of large tyrannosaurids in Laramidia, and is consistent with a late Campanian appearance of giant tyrannosaurins. Nevertheless, additional remains from the Kirtland, isolated teeth or bones, or ideally associated remains, are needed to better constrain both the size and the relationships of the Hunter Wash tyrannosaur.

Penecontemporaneous southern assemblages, e.g. the Kaiparowits Formation of Utah^22^, the Alto Shale of the Aguja Formation in Texas^40^, or the Cerro del Pueblo^15^ and El Gallo formations in Mexico^41^, could conceivably reveal additional evidence of large tyrannosaurs, either via further exploration in the field or restudy of existing museum collections. At the same time, the fact that large tyrannosaurids have remained unrecognized from the late Campanian until recently suggests that they were either rare members of these assemblages, and/or geographically restricted in their range. This is in striking contrast to the situation in the late Maastrichtian of Laramidia where *Tyrannosaurus* was widespread, ranging from New Mexico^42^ and Utah^43^ to the northern Great Plains^9,24^ and southern Canada^44,45^ and where *Tyrannosaurus* is the most abundant tyrannosaurid in terms of skeletons recovered.

The appearance of a large, derived tyrannosaurine in the late Campanian of the American Southwest also has implications for the geographic origin of *Tyrannosaurus*. Competing theories have favored an origin of *T*. *rex* either in Asia^2,11^, or in North America^13,14^, specifically in southern Laramidia^13^. While models have been put forth regarding the non-North American origins of tyrannosaurs^11^ this study shows that these models must account for this large, derived tyrannosaur from the Campanian of New Mexico.

**Fig. 6:**
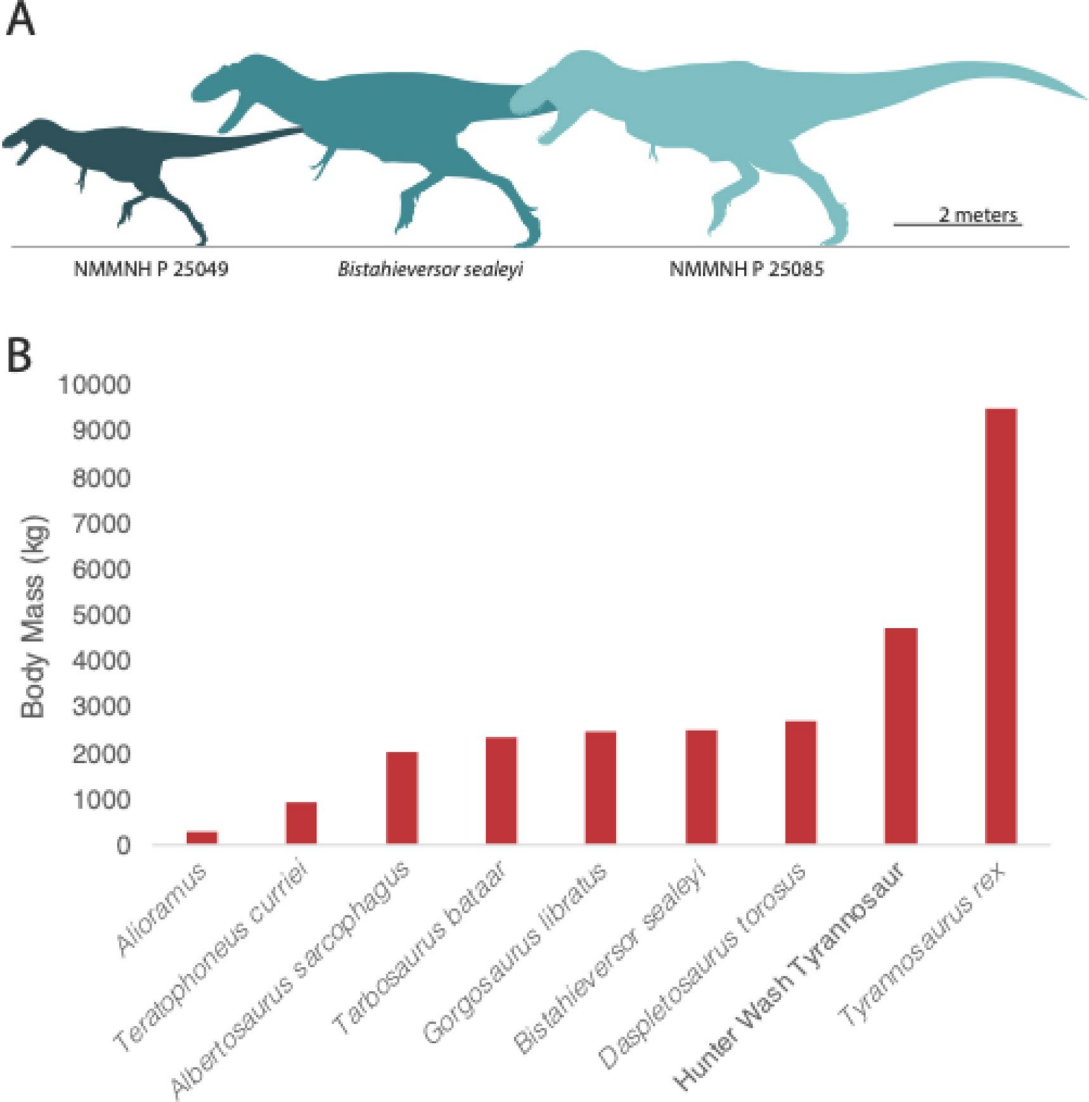
(**A**) Relative sizes of New Mexican Tyrannosauridae. NMMNH P 25085 is reconstructed as slightly longer than *Bistahieversor*, but its more robust construction suggests it was disproportionately massive. NMMNH P 25049 has previously been identified as a juvenile of *Bistahieversor* but might represent a distinct taxon of small-bodied tyrannosauroid. (**B**) body masses (kg) for Campanian and Maastrichtian Tyrannosauroidea^6,7^. The estimated mass of NMMNH P-25085 is higher than for any other contemporaneous tyrannosaur and approaches the mass of *Tyrannosaurus rex*.

The appearance of a giant tyrannosaur in the late Campanian of New Mexico, but not in more northern assemblages, is striking and emphasizes the high endemicity that existed during the late Campanian in Laramidia, as found previously^14,15,46,47^. While giant tyrannosaurs evolved in southern Laramidia, smaller tyrannosaurs, including Teratophoneini^14^, Albertosaurinae^23,25,48,49^, and Daspletosaurinae^12^ were apex predators in habitats such as the Kaiparowits Formation of Utah^14^, the Two Medicine Formation of Montana^12^, and the Dinosaur Park^48,49^ and Horseshoe Canyon Formations^23,25^ of Alberta (Fig. 6).

Not only did tyrannosaurs first achieve large size in the south, a number of other lineages evolved large size in southern Laramidia. These include chasmosaurine ceratopsians^46^, lambeosaurine^50^, saurolophines^51^ and titanosaurs^52,53^. Some lineages such as giant chasmosaurines appear to have become large in the south before dispersing north^46^ while others, including giant lambeosaurines and titanosaurs, were endemic to the south^50,52,53^. This suggests that not only did southern Laramidia have distinct species, but that they occupied distinct ecological niches and showed distinct evolutionary dynamics.

Finally, the Hunter Wash tyrannosaur hints at previously unrecognized diversity among southern tyrannosaurs, with *Bistahieversor* coexisting with a larger and more robust tyrannosaur species represented by NMMNH P-25085 (Fig. 6). It is possible that other tyrannosaurs existed in the Fruitland-Kirtland of New Mexico as well. A small tyrannosaur, NMMNH P-27469, has previously been identified as a juvenile of *Bistahieversor*^36^ but shows features seen in members of the Nanotyrannidae^5,37^ and other basal tyrannosauroids, including, e.g., a long anterior ramus of the maxilla, and a lateral groove of the dentary, which are not known in young Tyrannosauridae^54^. It also exhibits one feature (condyle of metatarsal III does not project anterior to metatarsal shaft) proposed as an autapomorphy of *Nanotyrannus*^5^. Further study of this specimen is needed, but in light of increasing evidence for high diversity of tyrannosaurs in the Late Maastrichtian^5,8^, it would be unsurprising if similar diversity existed in the Campanian.

The southern theropod assemblage therefore appears to have been more diverse than previously recognized. Further study of the Cretaceous dinosaur record in the Southwest seems likely to produce unexpected diversity and to shed light on the evolution of North American dinosaurs.

## Supplementary Information

Below is the link to the electronic supplementary material.

Supplementary Material 1

Supplementary Material 2

## Data Availability

All data generated or analyzed during this study are included in the manuscript or supporting information files.
