# Supplementary material for: A large tyrannosaurid from the Late Cretaceous (Campanian) of North America
Source: Sci Rep. 2026 Mar 12;16:8371. doi: 10.1038/s41598-026-38600-w (PMC12982495; doi:10.1038/s41598-026-38600-w)
Supplement: Supplementary file 2 — Supplementary Material 2 [file 41598_2026_38600_MOESM2_ESM.docx]

Equal Weights Parsimony

Tree length = 1925

Consistency index (CI) = 0.3673

Homoplasy index (HI) = 0.6327

CI excluding uninformative characters = 0.3669

HI excluding uninformative characters = 0.6331

Retention index (RI) = 0.7608

Rescaled consistency index (RC) = 0.2794

Strict consensus of 1404 trees:

/------------------------------------------------------------------ Tawa hallae

|

| /--------------------------------------------------------------- Coelophysis bauri

| |

\--+ /------------------------------------------------------------- Dilophosaurus wetherilli

| |

\-+ /---------------------------------------------------------- Ceratosaurus nasicornis

| |

\--+ /------------------------------------------------------- Dubreuillosaurus valesdunensis

| |

| | /----------------------------------------------------- Piatnitzkysaurus floresi

\--+ |

| | /-------------------------------------------------- Eustreptospondylus oxoniensis

| | |

\-+ | /----- Monolophosaurus jiangi

| | |

| | /------------------------------------------+ /--- Sinraptor dongi

| | | \-+

\--+ | \--- Allosaurus fragilis

| |

| | /--------------------------------------------- Tanycolagreus topwilsoni

| | |

| | | /----- Coelurus fragilis

| | | |

\-+ | /----------+ /--- Sinosauropteryx prima

| | | \-+

| | | \--- Compsognathus longipes

| | |

| | | /-------- Ornitholestes hermanni

| | /-------------------------+ |

| | | | | /--- Pelecanimimus polydon

| | | | /----+ /-+

\--+ | | | | | \--- Gallimimus bullatus

| | | | \--+

| | | | | /--- Shenzhousaurus orientalis

| | \--+ \-+

| | | \--- Harpymimus okladnikovi

| | |

| | | /----------- Haplocheirus sollers

| | | |

| | \-+ /-------- Archaeopteryx lithographica

| | | |

| | | | /--- Utahraptor ostrommaysorum

| | \--+ /-+

\--+ | | \--- Deinonychus antirrhopus

| \--+

| | /--- Linheraptor exquisitus

| \-+

| \--- Velociraptor mongolienesis

|

| /---------------------------------------- Proceratosaurus bradleyi

| |

| +---------------------------------------- Kileskus aristotocus

| |

| +---------------------------------------- Guanlong wucaii

| |

| +---------------------------------------- Dilong paradoxus

| |

\-+---------------------------------------- Eotyrannus lengi

|

+---------------------------------------- Juratyrant langhami

|

| /------------------------------------- Yutyrannus huali

| |

| | /---------------------------------- Dryptosaurus aquilunguis

\--+ |

| | /-------------------------------- Xiongguanlong baimoensis

| | |

\--+ +-------------------------------- Alectrosaurus olseni

| |

| +-------------------------------- Jinbeisaurus wangi

| |

| +-------------------------------- Suskityrannus hazelae

\-+

| /----------------------------- Appalachiosaurus montgomeriensis

| |

| | /--- Alioramus remotus

| | |

| | /----------------------+--- Alioramus altai

\--+ | |

| | \--- Qianzhousaurus sinensis

| |

| | /----------- Khaankhuuluu mongoliensis

| | |

\--+ /------------+ /-------- Nanotyrannus lancensis

| | | |

| | \--+ /----- Stygivenator lethaeus

| | | |

| | \--+ /--- Bloody Mary

| | \-+

| | \--- Stygivenator

\-+

| /--- Gorgosaurus libratus

| /-----------------+

| | \--- Albertosaurus sarcophagus

| |

| | /------------------ Asiatyrannus

\--+ |

| | /-------- Thanatotheristes degrootorum

| | |

| | /-------+ /----- Daspletosaurus horneri

\--+ | | |

| | \--+ /--- Daspletosaurus torosus

| | \-+

| | \--- Daspletosaurus wilsoni

| |

| | /-------- Dynamoterror dynastes

\-+ |

| +-------- Teratophoneus curriei

| /----+

| | | /----- Bistahieversor sealeyi

| | | |

| | \--+ /--- Labocania anomala

| | \-+

\--+ \--- Labocania aguilloni

|

| /----------- Lythronax argestes

| |

\-+ /-------- Nanuqsaurus hoglundi

| |

| | /--- Zhuchengtyrannus magnus

\--+ /-+

| | \--- Tarbosaurus bataar

| |

\--+ /--- Tyrannosaurus mcraeensis

| |

\-+--- Tyrannosaurus rex

|

\--- Hunter Wash tyrannosaurine

Goloboff fit = -354.45702 (tree length = 1928)

Consistency index (CI) = 0.3667

Homoplasy index (HI) = 0.6333

CI excluding uninformative characters = 0.3664

HI excluding uninformative characters = 0.6336

Retention index (RI) = 0.7602

Rescaled consistency index (RC) = 0.2788

Strict consensus of 360 trees:

/--------------------------------------------------------------------- Tawa hallae

|

| /------------------------------------------------------------------- Coelophysis bauri

| |

\-+ /---------------------------------------------------------------- Dilophosaurus wetherilli

| |

\--+ /-------------------------------------------------------------- Ceratosaurus nasicornis

| |

\-+ /----------------------------------------------------------- Dubreuillosaurus valesdunensis

| |

| | /--------------------------------------------------------- Piatnitzkysaurus floresi

\--+ |

| | /------------------------------------------------------ Eustreptospondylus oxoniensis

| | |

\-+ | /----- Monolophosaurus jiangi

| | |

| | /----------------------------------------------+ /-- Sinraptor dongi

| | | \--+

\--+ | \-- Allosaurus fragilis

| |

| | /------------------------------------------------- Tanycolagreus topwilsoni

| | |

| | | /----- Coelurus fragilis

\-+ | |

| | /-----------+ /-- Sinosauropteryx prima

| | | \--+

| | | \-- Compsognathus longipes

| | |

| | | /------- Pelecanimimus polydon

| | /-----------------------------+ |

| | | | /-------+ /----- Shenzhousaurus orientalis

\--+ | | | | |

| | | | \-+ /-- Harpymimus okladnikovi

| | | | \--+

| | \-+ \-- Gallimimus bullatus

| | |

| | | /------------ Ornitholestes hermanni

| | | |

| | \--+ /---------- Haplocheirus sollers

| | | |

| | \-+ /------- Archaeopteryx lithographica

| | | |

| | | | /-- Utahraptor ostrommaysorum

\-+ \--+ /--+

| | | \-- Deinonychus antirrhopus

| \-+

| | /-- Linheraptor exquisitus

| \--+

| \-- Velociraptor mongolienesis

|

| /-- Proceratosaurus bradleyi

| |

| +-- Kileskus aristotocus

| /-----------------------------------------+

| | +-- Guanlong wucaii

| | |

| | \-- Juratyrant langhami

\--+

| /------------------------------------------ Dilong paradoxus

| |

\-+ /--------------------------------------- Eotyrannus lengi

| |

\--+ /------------------------------------- Yutyrannus huali

| |

\-+ /---------------------------------- Dryptosaurus aquilunguis

| |

| | /-- Xiongguanlong baimoensis

\--+ /-----------------------------+

| | \-- Alectrosaurus olseni

| |

\-+ /------------------------------ Suskityrannus hazelae

| |

| | /--------------------------- Jinbeisaurus wangi

\-+ |

| | /------------------------- Appalachiosaurus montgomeriensis

| | |

\--+ | /-- Alioramus remotus

| | |

| | /-------------------+-- Alioramus altai

\-+ | |

| | \-- Qianzhousaurus sinensis

| |

| | /---------- Khaankhuuluu mongoliensis

| | |

\--+ /---------+ /------- Nanotyrannus lancensis

| | | |

| | \--+ /----- Stygivenator lethaeus

| | | |

| | \-+ /-- Bloody Mary

| | \--+

| | \-- Stygivenator

\-+

| /-- Gorgosaurus libratus

| /--------------+

| | \-- Albertosaurus sarcophagus

| |

| | /--------------- Asiatyrannus

\--+ |

| | /------- Thanatotheristes degrootorum

| | |

| | /----+ /----- Daspletosaurus horneri

\-+ | | |

| | \-+ /-- Daspletosaurus torosus

| | \--+

| | \-- Daspletosaurus wilsoni

| |

| | /---------- Dynamoterror dynastes

\--+ |

| +---------- Nanuqsaurus hoglundi

| |

| +---------- Teratophoneus curriei

| |

| | /----- Bistahieversor sealeyi

\-+ |

+----+ /-- Labocania anomala

| \--+

| \-- Labocania aguilloni

|

| /------- Lythronax argestes

| |

| | /-- Zhuchengtyrannus magnus

\--+ /--+

| | \-- Tarbosaurus bataar

| |

\-+ /-- Tyrannosaurus mcraeensis

| |

\--+-- Tyrannosaurus rex

|

\-- Hunter Wash tyrannosaurine

Goloboff fit = -423.92057 (tree length = 1928)

Consistency index (CI) = 0.3667

Homoplasy index (HI) = 0.6333

CI excluding uninformative characters = 0.3664

HI excluding uninformative characters = 0.6336

Retention index (RI) = 0.7602

Rescaled consistency index (RC) = 0.2788

Strict consensus of 360 trees:

/--------------------------------------------------------------------- Tawa hallae

|

| /------------------------------------------------------------------- Coelophysis bauri

| |

\-+ /---------------------------------------------------------------- Dilophosaurus wetherilli

| |

\--+ /-------------------------------------------------------------- Ceratosaurus nasicornis

| |

\-+ /----------------------------------------------------------- Dubreuillosaurus valesdunensis

| |

| | /--------------------------------------------------------- Piatnitzkysaurus floresi

\--+ |

| | /------------------------------------------------------ Eustreptospondylus oxoniensis

| | |

\-+ | /----- Monolophosaurus jiangi

| | |

| | /----------------------------------------------+ /-- Sinraptor dongi

| | | \--+

\--+ | \-- Allosaurus fragilis

| |

| | /------------------------------------------------- Tanycolagreus topwilsoni

| | |

| | | /----- Coelurus fragilis

\-+ | |

| | /-----------+ /-- Sinosauropteryx prima

| | | \--+

| | | \-- Compsognathus longipes

| | |

| | | /------- Pelecanimimus polydon

| | /-----------------------------+ |

| | | | /-------+ /----- Shenzhousaurus orientalis

\--+ | | | | |

| | | | \-+ /-- Harpymimus okladnikovi

| | | | \--+

| | \-+ \-- Gallimimus bullatus

| | |

| | | /------------ Ornitholestes hermanni

| | | |

| | \--+ /---------- Haplocheirus sollers

| | | |

| | \-+ /------- Archaeopteryx lithographica

| | | |

| | | | /-- Utahraptor ostrommaysorum

\-+ \--+ /--+

| | | \-- Deinonychus antirrhopus

| \-+

| | /-- Linheraptor exquisitus

| \--+

| \-- Velociraptor mongolienesis

|

| /-- Proceratosaurus bradleyi

| |

| +-- Kileskus aristotocus

| /-----------------------------------------+

| | +-- Guanlong wucaii

| | |

| | \-- Juratyrant langhami

\--+

| /------------------------------------------ Dilong paradoxus

| |

\-+ /--------------------------------------- Eotyrannus lengi

| |

\--+ /------------------------------------- Yutyrannus huali

| |

\-+ /---------------------------------- Dryptosaurus aquilunguis

| |

| | /-- Xiongguanlong baimoensis

\--+ /-----------------------------+

| | \-- Alectrosaurus olseni

| |

\-+ /------------------------------ Suskityrannus hazelae

| |

| | /--------------------------- Jinbeisaurus wangi

\-+ |

| | /------------------------- Appalachiosaurus montgomeriensis

| | |

\--+ | /-- Alioramus remotus

| | |

| | /-------------------+-- Alioramus altai

\-+ | |

| | \-- Qianzhousaurus sinensis

| |

| | /---------- Khaankhuuluu mongoliensis

| | |

\--+ /---------+ /------- Nanotyrannus lancensis

| | | |

| | \--+ /----- Stygivenator lethaeus

| | | |

| | \-+ /-- Bloody Mary

| | \--+

| | \-- Stygivenator

\-+

| /-- Gorgosaurus libratus

| /--------------+

| | \-- Albertosaurus sarcophagus

| |

| | /--------------- Asiatyrannus

\--+ |

| | /------- Thanatotheristes degrootorum

| | |

| | /----+ /----- Daspletosaurus horneri

\-+ | | |

| | \-+ /-- Daspletosaurus torosus

| | \--+

| | \-- Daspletosaurus wilsoni

| |

| | /---------- Dynamoterror dynastes

\--+ |

| +---------- Nanuqsaurus hoglundi

| |

| +---------- Teratophoneus curriei

| |

| | /----- Bistahieversor sealeyi

\-+ |

+----+ /-- Labocania anomala

| \--+

| \-- Labocania aguilloni

|

| /------- Lythronax argestes

| |

| | /-- Zhuchengtyrannus magnus

\--+ /--+

| | \-- Tarbosaurus bataar

| |

\-+ /-- Tyrannosaurus mcraeensis

| |

\--+-- Tyrannosaurus rex

|

\-- Hunter Wash tyrannosaurine

Goloboff fit = -463.36848 (tree length = 1926)

Consistency index (CI) = 0.3671

Homoplasy index (HI) = 0.6329

CI excluding uninformative characters = 0.3668

HI excluding uninformative characters = 0.6332

Retention index (RI) = 0.7606

Rescaled consistency index (RC) = 0.2792

Strict consensus of 135 trees:

/--------------------------------------------------------------------- Tawa hallae

|

| /------------------------------------------------------------------- Coelophysis bauri

| |

\-+ /---------------------------------------------------------------- Dilophosaurus wetherilli

| |

\--+ /-------------------------------------------------------------- Ceratosaurus nasicornis

| |

\-+ /----------------------------------------------------------- Dubreuillosaurus valesdunensis

| |

| | /--------------------------------------------------------- Piatnitzkysaurus floresi

\--+ |

| | /------------------------------------------------------- Eustreptospondylus oxoniensis

| | |

\-+ | /----- Monolophosaurus jiangi

| | |

| | /----------------------------------------------+ /-- Sinraptor dongi

| | | \--+

\-+ | \-- Allosaurus fragilis

| |

| | /-------------------------------------------------- Tanycolagreus topwilsoni

| | |

| | | /----- Coelurus fragilis

\--+ | |

| | /--------+ /-- Sinosauropteryx prima

| | | \--+

| | | \-- Compsognathus longipes

| | |

| | | /---------- Ornitholestes hermanni

| | /---------------------------------+ |

| | | | /-+ /------- Pelecanimimus polydon

\-+ | | | | |

| | | | \--+ /----- Shenzhousaurus orientalis

| | | | | |

| | | | \-+ /-- Harpymimus okladnikovi

| | \-+ \--+

| | | \-- Gallimimus bullatus

| | |

| | | /---------- Haplocheirus sollers

| | | |

| | \-+ /------- Archaeopteryx lithographica

| | | |

| | | | /-- Utahraptor ostrommaysorum

\-+ \--+ /--+

| | | \-- Deinonychus antirrhopus

| \-+

| | /-- Linheraptor exquisitus

| \--+

| \-- Velociraptor mongolienesis

|

| /-- Proceratosaurus bradleyi

| |

| +-- Kileskus aristotocus

| /------------------------------------------+

| | +-- Guanlong wucaii

| | |

| | \-- Juratyrant langhami

\--+

| /------------------------------------------- Dilong paradoxus

| |

\-+ /---------------------------------------- Eotyrannus lengi

| |

\--+ /-------------------------------------- Yutyrannus huali

| |

\-+ /------------------------------------ Dryptosaurus aquilunguis

| |

| | /-- Xiongguanlong baimoensis

\-+ /------------------------------+

| | \-- Alectrosaurus olseni

| |

\--+ /------------------------------- Suskityrannus hazelae

| |

| | /----------------------------- Jinbeisaurus wangi

\-+ |

| | /-------------------------- Appalachiosaurus montgomeriensis

| | |

\-+ | /-- Alioramus remotus

| | |

| | /---------------------+-- Alioramus altai

\--+ | |

| | \-- Qianzhousaurus sinensis

| |

| | /---------- Khaankhuuluu mongoliensis

| | |

\-+ /----------+ /------- Nanotyrannus lancensis

| | | |

| | \--+ /----- Stygivenator lethaeus

| | | |

| | \-+ /-- Bloody Mary

| | \--+

| | \-- Stygivenator

\--+

| /-- Gorgosaurus libratus

| /----------------+

| | \-- Albertosaurus sarcophagus

| |

| | /----------------- Asiatyrannus

\-+ |

| | /------- Thanatotheristes degrootorum

| | |

| | /------+ /----- Daspletosaurus horneri

\-+ | | |

| | \-+ /-- Daspletosaurus torosus

| | \--+

| | \-- Daspletosaurus wilsoni

| |

| | /------- Dynamoterror dynastes

\--+ |

| +------- Teratophoneus curriei

| /----+

| | | /----- Bistahieversor sealeyi

| | | |

| | \-+ /-- Labocania anomala

| | \--+

\-+ \-- Labocania aguilloni

|

| /---------- Lythronax argestes

| |

\-+ /------- Nanuqsaurus hoglundi

| |

| | /-- Zhuchengtyrannus magnus

\--+ /--+

| | \-- Tarbosaurus bataar

| |

\-+ /-- Tyrannosaurus mcraeensis

| |

\--+-- Tyrannosaurus rex

|

\-- Hunter Wash tyrannosaurine
